# Vanadium Accumulation and Reduction by Vanadium-Accumulating Bacteria Isolated from the Intestinal Contents of *Ciona robusta*

**DOI:** 10.1007/s10126-024-10300-4

**Published:** 2024-03-07

**Authors:** Dewi Yuliani, Fumihiro Morishita, Takuya Imamura, Tatsuya Ueki

**Affiliations:** 1 Laboratory of Molecular and Cellular Physiology, Graduate School of Integrated Sciences for Life, Hiroshima University, Higashi-Hiroshima, 1-3-1 Kagamiyama, Hiroshima, 739-8526 Japan; 2 Chemistry Department, Faculty of Mathematics and Natural Sciences, State Islamic University of Malang, Malang, 65145 Indonesia

**Keywords:** Ascidian, Intestinal bacteria, Ion exchange chromatography

## Abstract

The sea squirt *Ciona robusta* (formerly *Ciona intestinalis* type A) has been the subject of many interdisciplinary studies. Known as a vanadium-rich ascidian, *C. robusta* is an ideal model for exploring microbes associated with the ascidian and the roles of these microbes in vanadium accumulation and reduction. In this study, we discovered two bacterial strains that accumulate large amounts of vanadium, CD2-88 and CD2-102, which belong to the genera *Pseudoalteromonas* and *Vibrio*, respectively. The growth medium composition impacted vanadium uptake. Furthermore, pH was also an important factor in the accumulation and localization of vanadium. Most of the vanadium(V) accumulated by these bacteria was converted to less toxic vanadium(IV). Our results provide insights into vanadium accumulation and reduction by bacteria isolated from the ascidian *C. robusta* to further study the relations between ascidians and microbes and their possible applications for bioremediation or biomineralization.

**Supplementary Information:**

The online version contains supplementary material available at 10.1007/s10126-024-10300-4.

## Introduction

Ascidians, commonly called sea squirts, are sessile marine invertebrates that inhabit shallow ocean water and have a wide geographical distribution. Henze (1911) reported that the blood cells (vanadocytes) of the ascidian *Phallusia mammillata* contain unexpectedly high levels of vanadium. This unusual phenomenon prompted studies of how ascidians accumulate vanadium (reviewed in Michibata et al. 2003; Michibata and Ueki 2012; Ueki et al. 2015). Our group has extensively investigated vanadium levels and chemical states in a wide range of ascidian species, and has found that ascidians belonging to the Phlebobranchia can generally accumulate high levels of vanadium. The highest vanadium concentration has been found in *Ascidia gemmata* (350 mM in blood cells) (Michibata et al. 1991), which corresponds to 10^7^ times that in seawater (35 nM) (Collier 1984), and the accumulated vanadium is mostly reduced to the + 3 oxidation state (Hirata and Michibata 1991). While vanadium occurs in + 3 (V(III)), + 4 (V(IV)), and + 5 (V(V)) oxidation states in aqueous solution, V(V) is the most common and stable form (Imtiaz et al. 2015; Wnuk 2023). V(V) is an oxyanion and is usually taken up by cells via anion transporters or the transferrin pathway (Dingley et al. 1982) and reduced to V(IV) in the cytoplasm. This reduction process has been proposed to be mediated by reducing agents, such as glutathione or NADPH, in the cytoplasm (Kanamori et al. 1999). In the vanadocytes of ascidians, V(IV) has been shown to be transported to the acidic organelle, the vacuole, via the Nramp family divalent cation transporter (Ueki et al. 2011), and reduced again to V(III) and stored (Hirata and Michibata 1991; Nette et al. 1999, 2004; Ueki et al. 2002). We identified vanadium-binding proteins, vanabins, from vanadocytes and coelomic fluid of ascidians (Michibata et al. 2003; Trivedi et al. 2003; Yamaguchi et al. 2004; Yoshihara et al. 2005), which can act as V(V) reductase in the cytoplasm of vanadocytes (Kawakami et al. 2006, 2009; Ueki et al. 2014). However, vanadium is also found in other ascidian tissues. In particular, high concentrations have been reported in the intestine, 1.80 mM in *Ascidia sydneiensis samea* (Romaidi and Ueki 2016) and 0.6 mM in *Ciona intestinalis* (Hirata and Michibata 1991; Trivedi et al. 2003).

The bacteria in the ascidian intestine are closely related to physiological processes, nutrition, and metabolism of the host (Sommer and Bäckhed 2013; Yang et al. 2022). Several studies have also shown that intestinal bacteria from ascidians are able to accumulate and reduce vanadium. Antipov et al. (2000) discovered *Pseudomonas isachenkovii* from the intestinal contents of an ascidian worm, and found that it could resist vanadium toxicity after exposure to 6 g/L vanadate and used vanadium as an electron acceptor for anaerobic respiration. Two previous studies found that *Shewanella oneidensis* was able to grow in the presence of vanadate, using it as the sole electron acceptor, and could reduce vanadate V(V) to vanadyl V(IV) ions (Carpentier et al. 2003, 2005).

Numerous studies have reported that bacteria can accumulate metals as adaptive responses, as bacteria are constantly exposed to unfavorable environmental conditions, such as heavy metal exposure, but they are able to adapt through various protective mechanisms (Mathivanan et al. 2021). For example, *Aeromonas sobria* accumulates copper and nickel (Qurbani et al. 2022) while *Lactobacillus plantarum* accumulates cadmium and lead metals (Ameen et al. 2020). *Pseudomonas idrijaensis*, *Marinomonas communis*, and *Bacillus subtilis* can take up mercury, arsenic, and iron, respectively (Takeuchi et al. 2007; dos Reis et al. 2014; Bourdineaud et al. 2020). *Micrococcus luteus* and *Bacillus cohnii* have the potential to accumulate zinc, chromium, and cobalt (Benmalek and Fardeau 2016; Marzuki et al. 2021). Three marine bacteria, i.e., *Pseudomonas putida*, *Bacillus cereus*, and *Pseudomonas pseudoalcaligenes* (Shirdam et al. 2006), and *Halomonas* sp. (Ghazvini and Mashkani 2009) have been reported to accumulate vanadium.

Exposure to high levels of vanadium in the ascidian body triggers its accumulation by some resistant bacteria. Vanadium is present at different levels in the tissues of ascidians, e.g., 1.4 mM in the branchial sac and 1.8 mM in the intestine (Ueki et al. 2015). The ascidian intestine and intestinal contents are exposed to the external environment and harbor highly diverse bacterial communities that play a number of roles in maintenance and regulation of the physiology and environmental adaptation (Dishaw et al. 2012; Sommer and Bäckhed 2013). To determine the contributions of bacteria to accumulation of vanadium in ascidians, our group previously studied vanadium-accumulating bacteria in the intestinal contents of *A. sydneiensis samea*. Two bacteria, *Shewanella* strain S-RA-6 and *Vibrio* strain S-RA-4, were shown to take up large amounts of vanadium (Romaidi and Ueki 2016).

In the present study, we investigated the contributions of bacteria in another vanadium-accumulating ascidian, *Ciona robusta* (previously known as *Ciona intestinalis* type A). *C. robusta* accumulates a moderate level of vanadium (0.6 mM) (Michibata et al. 1991; Trivedi et al. 2003), which is 17,000 times higher than that in seawater. Although the gut microbiota of *Ciona* has been studied by several groups to examine the pivotal roles of the microbes in shaping the host immune system (Dishaw et al. 2012, 2014; Leigh et al. 2016; Utermann et al. 2020), there have been no previous reports on the relations between microbes and ascidian tissues in *C. robusta* with regard to vanadium accumulation. This prompted us to isolate and study cultivable bacteria associated with the intestinal contents of *C. robusta* to explore their vanadium accumulation ability.

## Materials and Methods

### Isolation of Vanadium-Resistant Bacteria

Adult *C. robusta* were obtained from the National BioResource Project (NBRP) of MEXT, Japan (https://marinebio.nbrp.jp/marinebio/). Bacterial strains were isolated at two different time points: soon after arriving at our laboratory and after 44 days in aquarium culture. *C. robusta* specimens were dissected aseptically, and the intestinal contents were collected in 1.5-mL microtubes. The contents were diluted from 10^−2^ to 10^−4^ with sterile natural seawater and 10-µL aliquots were inoculated onto agar medium. Three different solid media were prepared in artificial seawater (ASW) (Marine Art SF1; Tomita Pharmaceutical, Kumamoto, Japan): standard medium (yeast extract 2.5 g/L, peptone 5.0 g/L, glucose 1.0 g/L, and agar 15.0 g/L) (Atlas 2005), 1/2TZ medium (yeast extract 0.50 g/L, peptone 2.5 g/L, HEPES 4.8 g/L, MnCl_2_·4H_2_O 0.20 g/L, and agar 15.0 g/L) (Maruyama et al. 1993), and Postgate’s B (PB) medium (yeast extract 0.20 g/L, sodium lactate 2.5 g/L, KH_2_PO_4_ 0.25 g/L, NH_4_Cl 0.5 g/L, and agar 15.0 g/L) (Antipov et al. 1998). Each medium was supplemented with 10 mM sodium orthovanadate (Sigma-Aldrich, St. Louis. MO, USA) and incubated at 20 °C for 2–4 days. Growing bacterial colonies were randomly selected for further identification.

### Identification of Bacterial Strains

Molecular identification of the selected bacterial strains was performed using the 16S ribosomal RNA (rRNA) gene sequencing method with the primers 306F 5′-CCAGACTCCTACGGGAGGCAGC-3′ and 935R 5′-CGAATTAAACCACATGCTCAAC-3′. For polymerase chain reaction (PCR), an appropriate number of bacterial cells picked up from each colony and 1 µM of each primer were mixed with PCR Master Mix (KAPA2G Robust HS ReadyMix; Kapa Biosystems, Wilmington, MA, USA) in a 10 µL reaction volume. The amount of DNA template corresponded to about 5–30 ng. PCR was performed for 30 cycles of 96 °C for 30 s, 50 °C for 40 s, and 72 °C for 40 s. The PCR product (~ 650 bp) was purified using a FastGene™ Gel/PCR Extraction Kit (Nippon Genetics, Tokyo, Japan). DNAs were sent to Fasmac Co. Ltd. (Kanagawa, Japan) to determine the DNA sequence by the Sanger sequencing method. The sequence data were compared to the nucleotide sequences in the NCBI database using BLASTn (https://blast.ncbi.nlm.nih.gov/). The sequences were aligned, and phylogenetic analysis was conducted using ClustalW (https://www.genome.jp/tools-bin/clustalw). The phylogenetic tree was visualized using Interactive Tree of Life (https://itol.embl.de/).

### Bioaccumulation Assay of the Vanadium-Resistant Bacteria

Bacterial cells in the log phase were used as an inoculum and cultured in standard medium with shaking at 180 rpm, for 24 h, at 25 °C. The medium used was supplemented with 0.5 mM V(V) or V(IV). The cells were harvested by centrifugation at 3500 rpm and 4 °C for 15 min and washed three times with vanadium-free medium. The pellets were dried overnight at 65 °C, mixed with 500 µL of 1 N nitric acid, and then heated overnight at 65 °C. Each sample was centrifuged at 10,000 rpm for 10 min and the vanadium concentration in the supernatant was measured by atomic absorption spectrometry (AAS) (Shirdam et al. 2006; Romaidi and Ueki 2016). 1/2TZ medium was also used as a comparative medium. Vanadium was expressed as nanograms per milligram dry weight (ng/mg dw) of cells.

### Accumulation of Vanadium at Different pH Values

Vanadium accumulation was determined according to variation in pH following the procedure described previously (Romaidi and Ueki 2016). Bacterial cells were prepared by culturing an inoculum in standard medium with rotation at 180 rpm and 25 °C for 24 h. The culture was centrifuged at 3500 rpm and 4 °C for 15 min for harvesting. Harvested cells were suspended in assay buffer (0.5 M NaCl, 0.05 mM sodium phosphate at pH 3, 5, 7, or 9) supplemented with 0.5 mM vanadium. The bacterial cell suspensions were rotated at 180 rpm and 25 °C for 24 h. The cells were collected by centrifugation and washed three times with assay buffer at the appropriate pH. After drying overnight at 65 °C, 0.5 mL 1 N nitric acid was added to the cells and incubated overnight at 65 °C. The mixture was centrifuged at 10,000 rpm for 10 min to obtain the supernatant. Vanadium accumulation was assessed by AAS.

### Subcellular Distribution of Vanadium in the Bacterial Cells

The distributions of vanadium in the intracellular and extracellular compartments of bacterial cells were determined according to the previously published methods with some modifications (Desaunay and Martins 2014; Romaidi and Ueki 2016). Bacterial cells were cultured in vanadium-free standard medium for 24 h at 25 °C. Then, cell pellets were incubated in fresh medium containing 50 mM phosphate buffer at the appropriate pH, 0.5 M NaCl, and 0.5 mM vanadium at 25 °C with agitation at 180 rpm. After 24 h of incubation, the bacterial pellets were resuspended with 20 mM EDTA and shaken gently for 5 min to remove vanadium bound to the outer membrane and associated with the periplasmic space. These suspensions were centrifuged at 3500 rpm for 10 min. The supernatant was used directly to assay vanadium in the extracellular compartment. The pellet was treated with 0.5 mM HNO_3_ and incubated at 90 °C overnight before AAS to assess vanadium in the intracellular compartment.

### Vanadium Speciation Assay Using Ion Exchange Chromatography

The ratio of V(V) to V(IV) in bacteria was examined by ion exchange chromatography, as described previously (Ueki et al. 2014). Overnight bacterial cultures were grown in standard medium supplemented with 0.5 mM V(V) at 25 °C and 180 rpm overnight. Harvested bacterial cells were washed with vanadium-free standard medium in triplicate. The bacterial cell pellet was resuspended with IC buffer (12 mM sodium hydrogen carbonate, 4 mM sodium carbonate, and 20 mM EDTA), and the mixture was sonicated to disrupt the cells. After centrifugation (10,000 rpm, 30 min), the pellet was discarded and the supernatant was analyzed for vanadium. Samples were injected into an ion exchange column (SI-50G; Shodex, Munich, Germany), equilibrated with IC buffer at a flow rate of 0.7 mL/min. Vanadium ions were detected by ultraviolet (UV) absorption at 282 nm and recorded with a D-7500 integrator (Hitachi, Ibaraki, Japan). The relative amounts of V(V) and V(IV) were calculated as the relative area ratio calculated from each peak profile.

## Results

### Isolation and Identification of Vanadium-Resistant Bacteria

We used culture medium containing 10 mM sodium orthovanadate (Na_3_VO_4_), which restricted the growth of intestinal bacteria in a previous study (Romaidi and Ueki 2016). We isolated bacteria from the intestinal contents of *C. robusta* at two different time points, i.e., immediately after arriving at the laboratory from NBRP or after 44 days in culture in the aquarium. We collected 40 bacterial colonies at random from the three different cultivation media; 9 isolates were obtained from the first isolation, and 31 isolates were obtained from the second isolation. All isolates were categorized as vanadium-resistant bacteria because of their ability to grow on medium supplemented with 10 mM sodium orthovanadate.

**Fig. 1:**
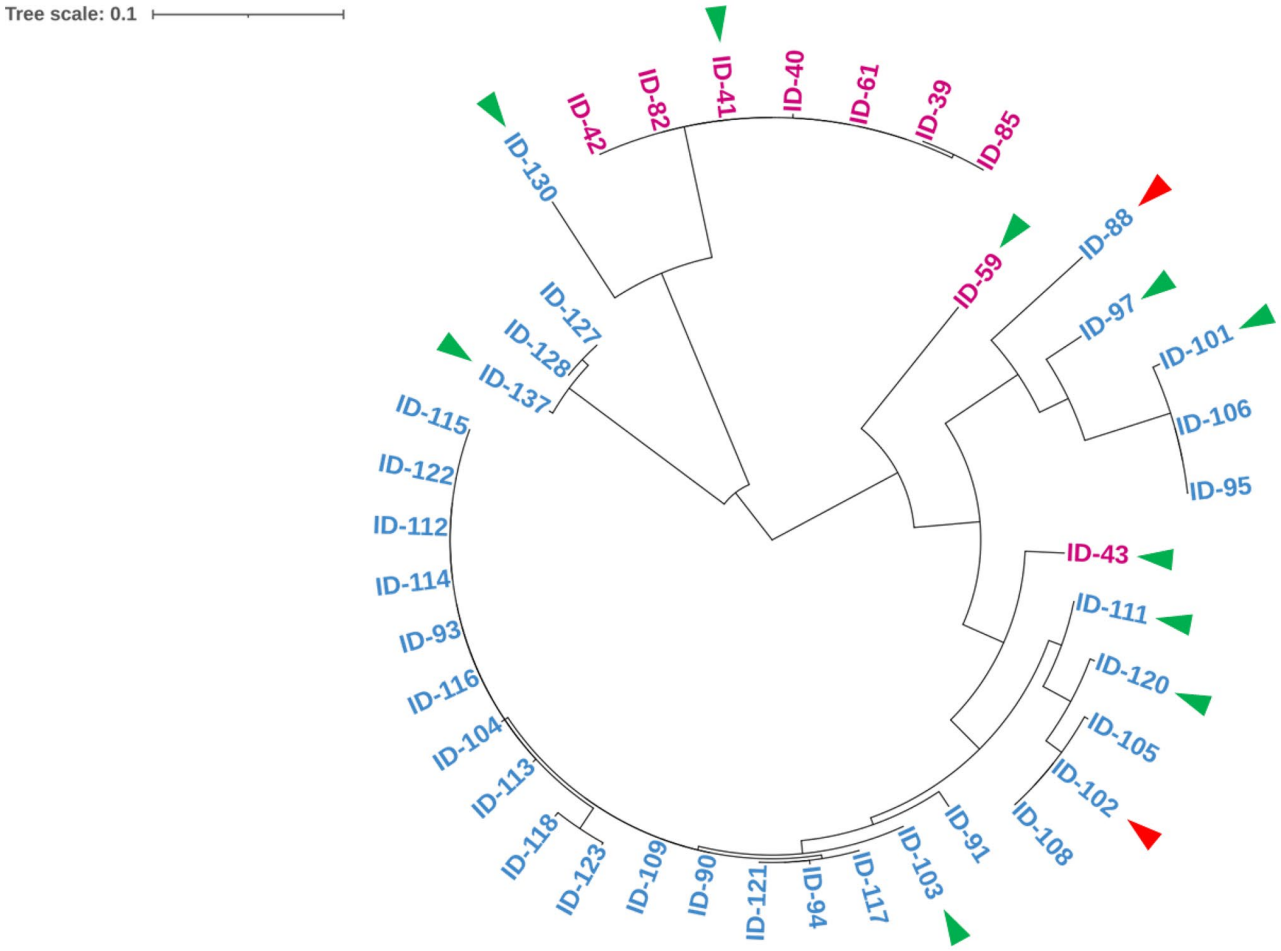
Phylogenetic tree of vanadium-resistant bacteria from the intestinal contents of *C. robusta* based on 16S rRNA sequences. After arriving at the laboratory (purple) or after treatment for 44 days (blue), we selected strains (green arrowheads) and identified hyperaccumulator vanadium strains (red arrowheads)

The 40 bacterial isolates were identified by partial 16S rRNA gene sequencing and could be classified into 2 phyla, 8 genera, and 12 strains (Supplementary Table S1). The 40 bacterial isolates formed two main clusters in phylogenetic tree analysis, which suggested that the types of culturable bacteria were different between the first and second isolation methods (Fig. 1). Thirty isolates belonged to the Proteobacteria and were composed of 72% Gammaproteobacteria and 8% Alphaproteobacteria, and eight isolates belonged to Firmicutes. At the genus level, the isolated bacteria were assigned to eight genera: *Vibrio* (58%), *Staphylococcus* (18%), *Shewanella* (8%), *Ensifer* (8%), *Photobacterium* (2%), *Pseudoalteromonas* (2%), *Ferrimonas* (2%), and *Halobacillus* (2%). Twelve isolates representing the 12 strains were selected (Table 1) and used to measure their ability to accumulate vanadium.

**Table 1:** Twelve vanadium-resistant bacteria isolated from the intestinal contents of *Ciona robusta*

**Bacterial strain**	**Related species**	**% similarity**
Isolation I		
CR1-41	*Staphylococcus ureilyticus* strain CK27	99.66
CR1-43	*Photobacterium damselae* strain NBRC 15633	98.83
CR1-59	*Vibrio aerogenes* strain FG1	90.00
Isolation II		
CD2-88	*Pseudoalteromonas phenolica* strain JCM 21460	98.96
CD2-97	*Ferrimonas kyonanensis* strain NBRC 101286	99.33
CD2-102	*Vibrio mediterranei* strain LMG 19703	99.16
CD2-103	*Vibrio alginolyticus* strain NBRC 15630	99.48
CD2-106	*Shewanella loihica* strain PV-4	99.16
CD2-111	*Vibrio neptunius* strain LMG 20536	99.49
CD2-120	*Vibrio diabolicus* strain HE800	99.16
CD2-130	*Halobacillus yeomjeoni* strain MSS-402	98.83
CD2-137	*Ensifer alkalisoli* strain YIC4027	94.06

The most culturable strain obtained (23 isolates) belonged to the genus *Vibrio*, corresponding closely to *Vibrio alginolyticus* (98.73% ± 1.31%), *Vibrio mediterranei* (99.32% ± 0.16%), *Vibrio diabolicus* (99.16%), *Vibrio neptunius* (99.49%), and *Vibrio aerogenes* (90.00%). The second most dominant strains (7 isolates) belonged to the genus *Staphylococcus* and were assigned to *Staphylococcus ureilyticus* with identity of 98.71% ± 1.08%. These were followed by the genera *Shewanella* and *Ensifer*. Three isolates (CD2-95, CD2-101, and CD2-106) showed 99.05% ± 0.34% similarity in 16S rRNA gene sequence to that of *Shewanella loihica*; three other isolates (CD2-127, CD2-128, and CD2-137) showed 94.01% ± 0.04% similarity to *Ensifer alkalisoli*. The remaining isolates, CR1-43 and CD2-97, showed 98.83% and 99.33% similarity to *Photobacterium damselae* and *Ferrimonas kyonanensis*, respectively, while CD2-130 showed 98.83% similarity to *Halobacillus marinus*.

### Accumulation Assay by the Vanadium-Resistant Bacteria

**Fig. 2:**
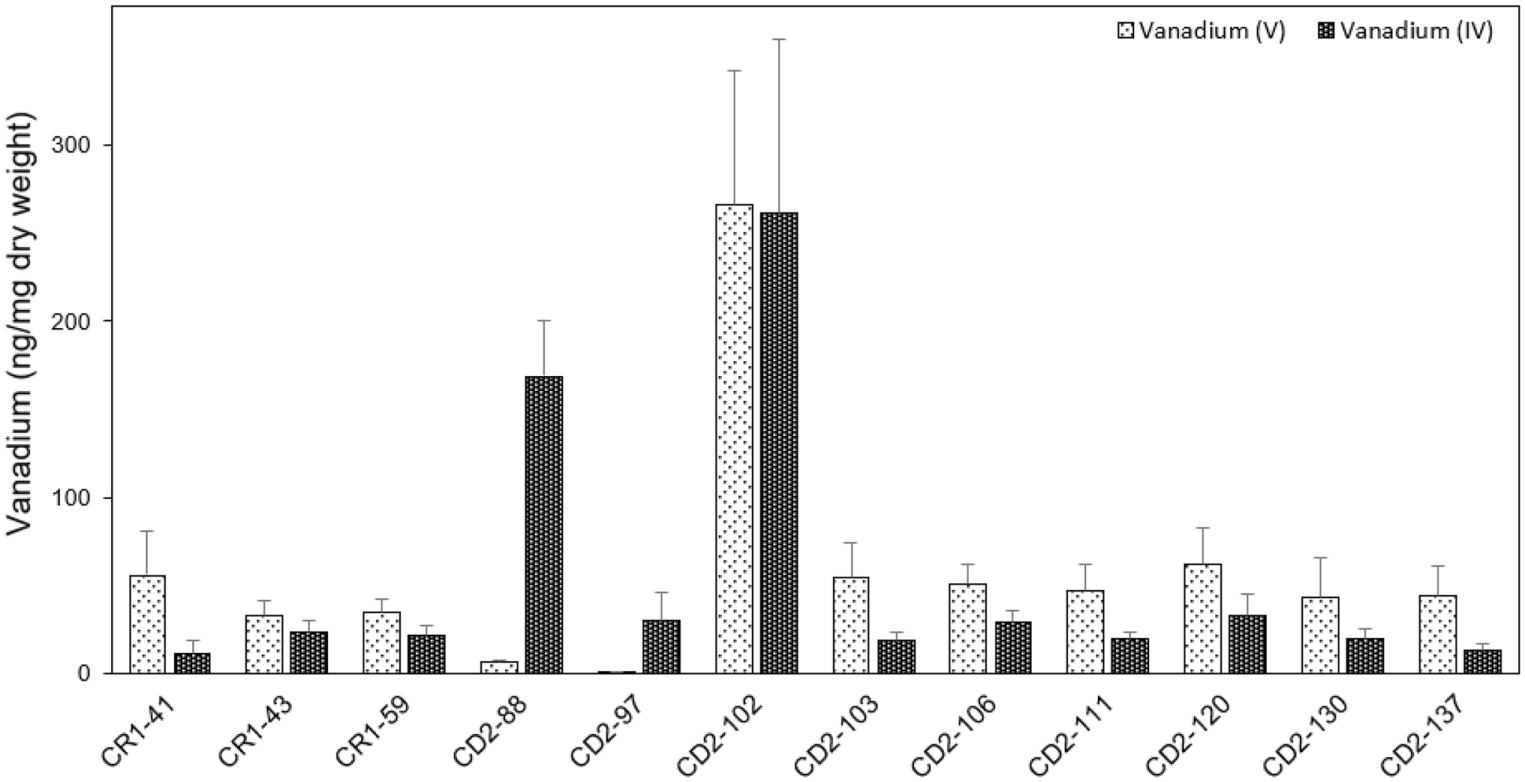
Accumulation of vanadium by selected vanadium-resistant bacterial strains isolated from the intestinal contents of *C. robusta*. Bacterial cells were cultured in standard medium supplemented with 0.5 mM V(V) or V(IV) for 24 h at 25 °C. The cells were harvested by centrifugation, and the amount of vanadium per dry weight of cells was measured. Error bars correspond to the standard deviation for 3–9 replicates

The abilities of the 12 strains to accumulate vanadium were evaluated by determining the V content per dw of bacterial cells in media containing 0.5 mM V(V) and V(IV). All strains accumulated different amounts of one or both vanadium species. However, only strains CD2-88 and CD2-102 showed high vanadium accumulation ability (Fig. 2). Two strains were potential vanadium hyperaccumulators and were used for further analyses. The vanadium accumulation abilities of these two strains, which were close to the genera *Pseudoalteromonas* and *Vibrio*, were examined in standard medium and 1/2TZ medium.

In the standard medium, strain CD2-102 accumulated 266 ± 77 ng/mg dw of V(V) and 261 ± 99 ng/mg dw of V(IV). No significant differences were observed between the accumulation of V(V) or V(IV) (Student’s *t* test, *p* > 0.05). The accumulation pattern of strain CD2-102 differed from that of strain CD2-88, which accumulated only a small amount of V(V) (6 ± 1 ng/mg dw) but a large amount of V(IV) (169 ± 31 ng/mg dw). The remaining strains accumulated various amounts of vanadium, ranging from 0.3 to 62 ng/mg dw for V(V) and from 11 to 32 ng/mg dw for V(IV).

**Fig. 3:**
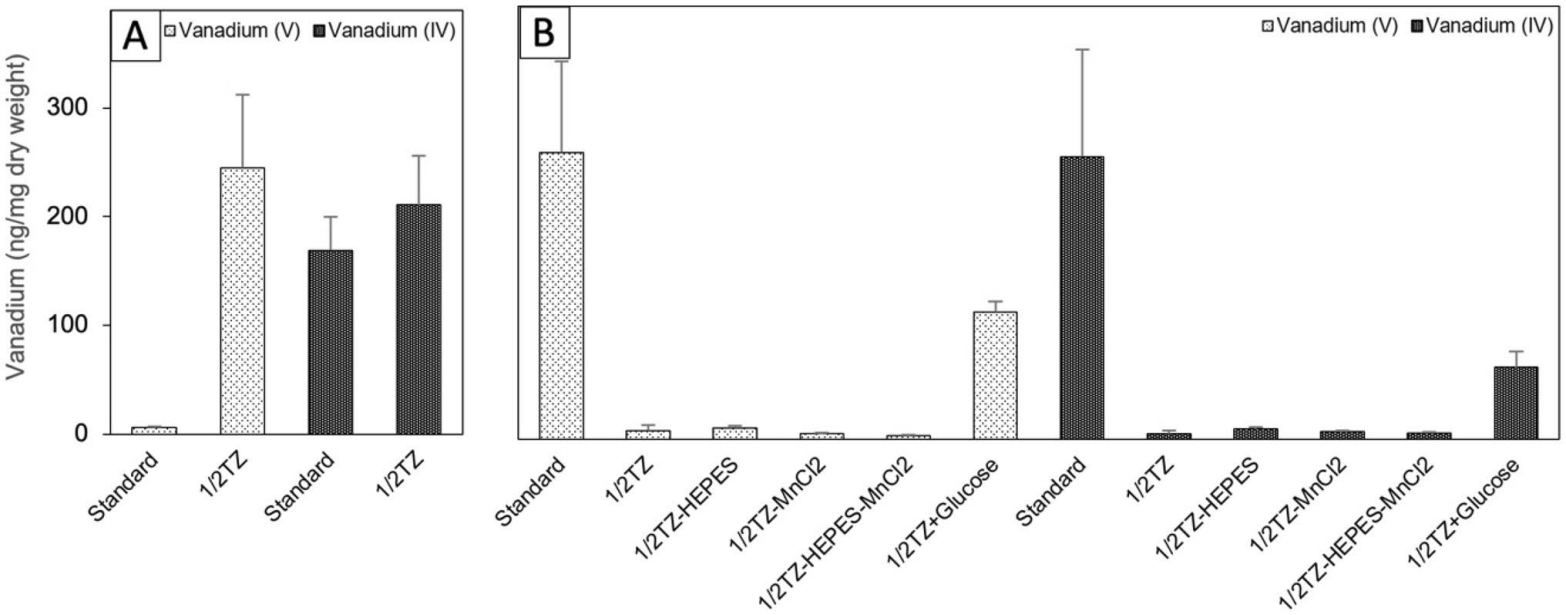
Accumulation of vanadium in the standard and 1/2TZ medium by **A** CD2-88 and **B** CD2-102. Bacterial cells were cultured in each medium supplemented with 0.5 mM V(V) or V(IV) for 24 h at 25 °C. The cells were harvested by centrifugation and the amount of vanadium per dry weight of cells was measured. The symbols − and + indicate removal and addition of substances to the medium, respectively. The error bars correspond to the standard deviation calculated from duplicate data for 3–7 replicates

We also investigated vanadium uptake in 1/2TZ medium, which affected the ability of the strains to accumulate vanadium. For strain CD2-88, 1/2TZ medium enhanced the vanadium uptake to 245 ± 67 ng/mg dw for V(V) and 211 ± 45 ng/mg dw for V(IV) (Fig. 3). This difference in V(V) accumulation was significant (*p* < 0.05), while that of V(IV) accumulation was not (*p* > 0.05). Interestingly, CD2-102 showed the opposite result, with vanadium accumulation capability decreasing by 98% to 8 ± 5 ng/mg dw for V(IV) and 5 ± 3 ng/mg dw for V(V). Next, we investigated the effects of altering the 1/2TZ medium composition on strain CD2-102. Removing the HEPES and/or manganese(II) chloride did not alter the accumulation ability. The amount of accumulated vanadium was still low at 6–9 ng/mg dw for V(IV) and 3–10 ng/mg dw for V(V) (Fig. 3). After adding glucose, vanadium accumulation increased significantly to 117 ± 10 ng/mg dw for V(V) and 66 ± 15 ng/mg dw for V(IV) (Student’s two-tailed *t* test).

### Vanadium Accumulation at Different pH Levels

**Fig. 4:**
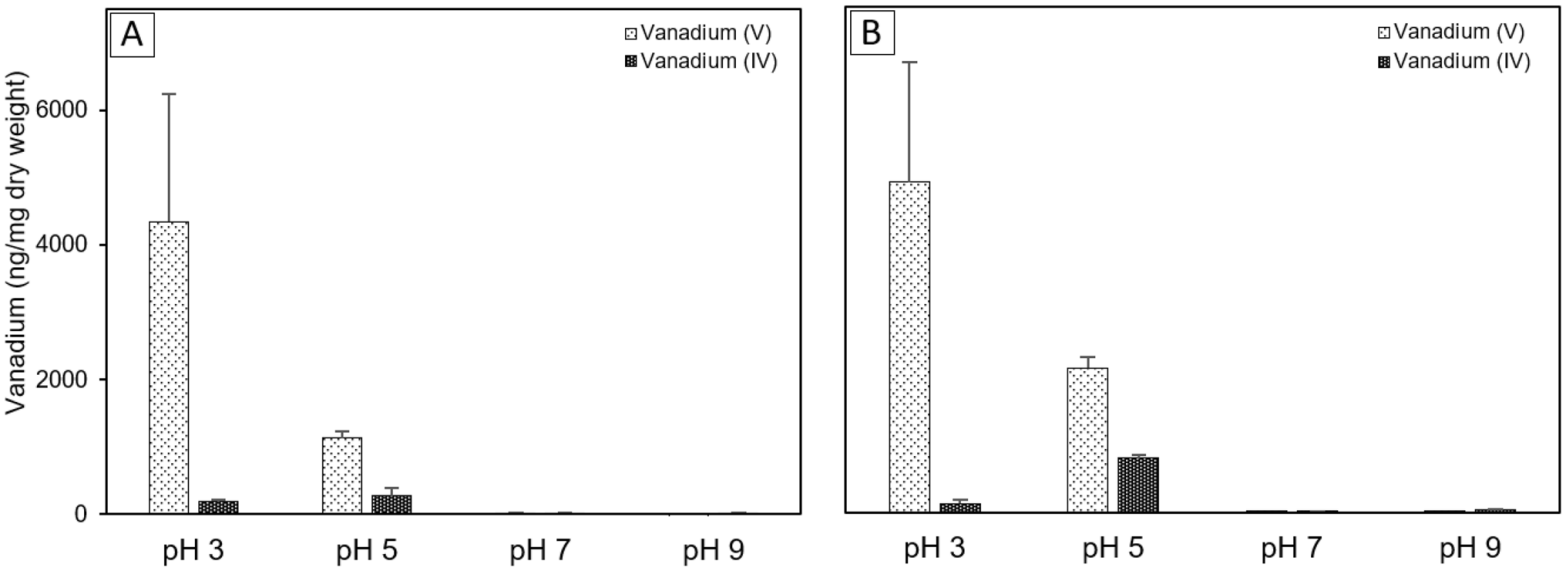
Effects of pH on vanadium accumulation in sodium chloride-sodium phosphate buffer (**A** CD2-88 and **B** CD2-102). Bacterial cells were cultured in standard medium, harvested by centrifugation, and resuspended in assay buffer (0.5 M NaCl, 0.05 mM sodium phosphate at pH 3, 5, 7, or 9) supplemented with 0.5 mM vanadium. The bacterial cell suspensions were incubated for 24 h at 25 °C. The cells were harvested by centrifugation and the amount of vanadium per dry weight of cells was measured. Vertical bars indicate the standard error (*n* = 3–6)

To study the influence of pH on vanadium accumulation, we cultured strains CD2-88 and CD2-102 in standard medium and harvested cells were incubated in an assay buffer at pH ranging from 3.0 to 9.0. Under these conditions, bacteria did not grow but could accumulate vanadium. The accumulation of vanadium was greatly affected by the pH (Student’s two-tailed *t* test; Fig. 4). The results showed that vanadium accumulation increased with decreasing pH. The greatest levels of V(V) accumulation by strains CD2-88 and CD2-102 were observed at pH 3 and were 4342 ± 1900 ng/mg dw and 4905 ± 1767 ng/mg dw, respectively. At pH 5, the V(V) concentration declined to 1132 ± 106 ng/mg dw for CD2-88 and 2150 ± 157 ng/mg dw for CD2-102. Under neutral conditions (pH 7), CD2-88 and CD2-102 took up 25 ± 4 ng/mg dw V(V) and 9 ± 2 ng/mg dw V(V), respectively. A negligible amount of V(V) was accumulated at pH 9. In strains CD2-88 and CD2-102, the V(IV) accumulation differed slightly from the V(V) accumulation pattern. The V(IV) uptake tended to increase with decreasing pH from pH 9.0 to 5.0, and then decreased at pH 3.0. The maximum capacities to accumulate V(IV) for CD2-88 and CD2-102 were 276 ± 110 ng/mg dw and 817 ± 44 ng/mg dw, respectively, which were observed at pH 5.0. At pH 3, the V(IV) accumulation decreased to 188 ± 26 ng/mg dw and 142 ± 49 ng/mg dw for CD2-88 and CD2-102, respectively. The V(IV) accumulation was low at pH 7 and 9. In general, the V(V) and V(IV) uptake were highest at acidic pH.

### Subcellular Localization of Vanadium in Bacterial Cells

**Fig. 5:**
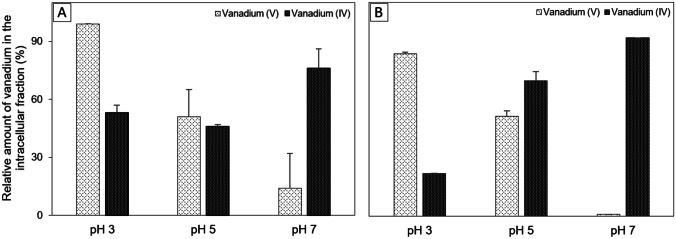
Relative amounts of vanadium in the intracellular fraction of **A** CD2-88 and **B** CD2-102. Bacterial cells were cultured in standard medium, harvested by centrifugation, and resuspended in assay buffer (0.5 M NaCl, 0.05 mM sodium phosphate at pH 3, 5, or 7) supplemented with 0.5 mM vanadium. The bacterial cell suspensions were incubated for 24 h at 25 °C. The cells were harvested by centrifugation, and the amount of vanadium per dry weight of cells was measured. Error bars correspond to the standard deviation (*n* = 3)

Experiments were performed to determine in which bacterial cell compartment the vanadium was localized after 24 h in culture. The amount of accumulated vanadium was measured at pH 3, 5, and 7. Bacteria can store metals on the cell surface (extracellular compartment) or inside the cell (intracellular compartment). The relative amount of vanadium was calculated by dividing that in the cytoplasm by the total amount in the cell. The pH and vanadium species greatly affected the localization of the metal in the cells (Fig. 5). At pH 7, the extracellular compartment contained as much as 87–99% of the vanadium, which decreased to 44–49% at pH 5 and 1–9% at pH 3. In contrast, at pH 7, the intracellular compartment contained 76–100% of the vanadium, which decreased to 46–76% and 24–53% at pH 5 and 3, respectively. Strains CD2-88 and CD2-102 showed similar patterns of subcellular localization of vanadium.

### Vanadium Speciation in Bacterial Cells

Ion chromatography was used to examine vanadium speciation in the cell. V(V) and V(IV) could be differentiated based on peak formation and retention time. The peak area was used to calculate the vanadium concentration. In this analysis, strains CD2-88 and CD2-102 were cultured on standard medium containing 0.5 mM V(V) as the sole vanadium species. A peak of V(IV) would appear if the V(V) in the medium was converted. We detected a large peak of V(IV) compared to V(V) in the bacterial cells. The percentage of V(IV) was 99.4% ± 0.007% for strain CD2-88 and 99.7% ± 0.003% for strain CD2-102, confirming that the bacteria reduced V(V) to V(IV).

## Discussion

We screened bacteria from the intestine of *C. robusta* for vanadium resistance by inoculating three culture media containing 10 mM sodium orthovanadate with aliquots of intestinal contents. Forty strains were obtained and were found to be resistant to vanadium.

*Vibrio* was the most culturable genus, accounting for 58% of all cultivated strains, and was considered one of the most abundant culturable bacteria present in the aquatic environment (Vezzulli et al. 2015) and marine animals (Sampaio et al. 2022). *Vibrio* has been reported to be a highly abundant bacterial genus in the gut of *Ciona* in general (Dishaw et al. 2012) and *Ciona intestinalis* in particular (Liberti et al. 2019). In addition to resistance to vanadium, some *Vibrio* species have also been shown to be resistant to other heavy metals. For example, *Vibrio parahaemolyticus* isolated from oysters and shellfish is resistant to barium, cobalt, cadmium, and copper (Kang et al. 2018; Jo et al. 2020) while *V. alginolyticus* has been reported to be resistant to arsenic (Takeuchi et al. 2007). Su et al. (2022) reported that *Vibrio* species from the clam *Meretrix meretrix* are tolerant to cadmium, zinc, and copper.

The second most dominant genus was *Staphylococcus*, which accounted for 18% of all cultivated strains, followed by other genera, such as *Shewanella*, *Ensifer*, *Photobacterium*, *Pseudoalteromonas*, *Ferrimonas*, and *Halobacillus*. These genera are commonly found in marine animals, such as ascidians (Dishaw et al. 2014), sponges (Fan et al. 2013; Paul et al. 2021), fish (Campbell et al. 2007), mussels (Beleneva and Maslennikova 2002; Collado et al. 2009), and sea urchins (Yao et al. 2019). *Shewanella* and *Pseudoalteromonas* have been reported to be prevalent components of the ascidian gut microbiome (Schreiber et al. 2016; Ueki et al. 2019). *Shewanella putrefaciens* and *Shewanella oneidensis* show significant resistance to arsenic, lead, and cadmium (Huang et al. 2011; Jaafar et al. 2016), whereas *Pseudoalteromonas citrea* and *Pseudoalteromonas nigrifaciens* show tolerance to copper, cadmium, mercury, and nickel (Ivanova et al. 2001). *Staphylococcus* shows resistance to arsenic, mercury, silver, and cadmium (Lauková 1994; Lawal et al. 2021) while *Ferrimonas kyonanensis*, *Photobacterium damselae*, and *Halobacillus* sp. have high tolerance to selenium, cadmium, and nickel, respectively (Nakagawa et al. 2006; Yakoubi et al. 2018; Kardel and Torabi 2019). We also obtained isolates belonging to *Ensifer*, a genus that was originally isolated from plant roots (Li et al. 2016) and shown to have arsenic resistance (Mesa et al. 2017; DiCenzo et al. 2018). No strains belonging to this genus have previously been isolated from marine animals, and this represents the first report of *Ensifer* isolated from an ascidian.

Two vanadium-resistant bacteria were identified as vanadium accumulators, i.e., CD2-88 (*Pseudoalteromonas* sp.) and CD2-102 (*Vibrio* sp.) (Fig. 2). *Pseudoalteromonas* shows good accumulation of cadmium (Zhou et al. 2013), and this species plays a predominant role in arsenic and lead remediation (Dell’Anno et al. 2020). To the best of our knowledge, however, there have been no previous reports of vanadium accumulation by this genus. In contrast, *Vibrio* sp. has been reported to be a bioremediation agent for heavy metals, including vanadium. For example, *Vibrio fluvialis* has the ability to take up mercury (Saranya et al. 2017) while *V. alginolyticus* and *Vibrio rotiferianus* have been shown to take up lead and strontium (Parmar et al. 2020). Our research group has also reported that *Vibrio* strain V-RA-4 can accumulate various metals, especially vanadium (Romaidi and Ueki 2016). Hernández et al. (1998) reported accumulation of high levels of vanadium, 35,004 ng/mg dw and 34,154 ng/mg dw, by *Escherichia hermannii* CNB50 and *Enterobacter cloacae* CNB60, respectively. The level of vanadium accumulation in this study was similar to that reported by Romaidi and Ueki (2016) but 100 times lower than reported by Hernández et al. (1998). The present study is the first report that *Pseudoalteromonas* species can accumulate vanadium, and we found more *Vibrio* sp. capable of accumulating vanadium.

In addition to accumulating vanadium, the strains CD2-88 and CD2-102 also reduced V(V) to V(IV). More than 99% of the V(V) taken up by both strains was converted into V(IV) in these bacterial cells. Various microorganisms have been reported to possess the ability to reduce V(V) to V(IV), such as *Pseudomonas* (Lyalkova and Yurkova 1992), *Shewanella* (Carpentier et al. 2003, 2005; Wang et al. 2017), *Geobacter* (Ortiz-Bernad et al. 2004; Liu et al. 2017; Yan et al. 2022), *Bacillus* (Zhang et al. 2019; Zhou et al. 2022), and *Polaromonas* (Sun et al. 2020). The metal-reducing bacterium *Shewanella oneidensis* can fully reduce 5 mM V(V) to support its growth (Carpentier et al 2003) while *E. cloacae* can reduce 55% of the V(V) at a vanadium concentration of 4 mM (van Marwijk et al. 2009). Here, we also found that our vanadium-resistant and vanadium-accumulating bacteria, *Pseudoalteromonas* sp. strain CD2-88 and *Vibrio* sp. strain CD2-102, could reduce vanadate V(V) to the lower oxidation state species V(IV). The capability of V(V) bioreduction by bacteria could differ between strains.

The type of growth medium may influence the metal accumulation capability of bacteria. The two strains were examined to determine optimal growth medium conditions, because such information will be necessary for their application to bioremediation. The standard medium and 1/2TZ medium are rich media and have been used to culture bacteria from marine sources (Yamazaki et al. 1993). Strain CD2-88 showed a low level of V(V) accumulation in standard medium, which surprisingly increased by 98% in 1/2TZ medium. In contrast, strain CD2-102 showed the opposite response upon changing to 1/2TZ medium, and addition of glucose to 1/2TZ medium led to 50% recovery of vanadium accumulation. The composition of the medium may have been one factor responsible for the decline in vanadium accumulation by strain CD2-102. This is supported by previous studies by Rathnayake et al. (2013) and El Baz et al. (2015) that medium composition impacts the level of metal tolerance. In this case, the addition of glucose partially restored the vanadium accumulation ability of CD2-102, which uses glucose as its primary source of energy. Bioaccumulation is a cellular energy–dependent process in active metabolic microorganisms (Wróbel et al. 2023). Our results parallel those of Stoll and Duncan (1996) and Bode et al. (1990) who demonstrated that pretreatment of *Saccharomyces cerevisiae* with glucose enhances heavy metal uptake from wastewater. Glucose is the most appropriate electron donor in the reduction of certain heavy metals (Zeng et al. 2019; Tan et al. 2020). Our results suggest that glucose could play an important role in vanadium accumulation by CD2-88 and CD2-102.

Accumulation is a complex process that depends on various factors, with pH being the most critical parameter in the accumulation of metal ions (Khan et al. 2016). Our results clearly show increasing bioaccumulation of vanadium by CD2-88 and CD2-102 with decreasing pH, with acidic pH 3 being the most favorable condition for vanadium accumulation. Similarly, Romaidi and Ueki (2016) reported optimal vanadium accumulation by *Vibrio* strain V-RA-4 and *Shewanella* strain S-RA-6 under acidic conditions. Bode et al. (1990) examined vanadium uptake by *S. cerevisiae* at low pH. In another study, both cadmium and copper biosorption by *Bacillus thuringiensis* OSM29 were optimized at low pH (Oves et al. 2013). In contrast, in another study, uptake of other metals was high at neutral and basic pH; e.g., accumulation of lead was maximized under neutral conditions, while chromium and nickel accumulation were maximized under basic conditions (López et al. 2000; Aslam et al. 2020). Taken together, these observations indicate that the influence of pH on metal accumulation varies among bacterial strains and metals.

We found changes in the distribution of V(V) and V(IV) in CD2-88 and CD2-102 strains according to pH. These two strains predominantly accumulated V(V) extracellularly at pH 7, shifting to intracellular storage at pH 3 (Fig. 5). Microorganisms can accumulate metals by either sorption on the cell surface or by intracellular uptake (Ledin 2000; Hansda et al. 2016). In a previous study, our group documented intracellular accumulation of vanadium by *Vibrio* strain V-RA-4 and *Shewanella* S-RA-6 (Romaidi and Ueki 2016). Almeida et al. (2020) support intracellular accumulation of vanadate by *Ochrobactrum tritici*. In contrast to previous studies, we documented both intracellular and extracellular vanadium uptake by bacterial strains isolated from the sea squirt *C. robusta*. Both accumulation processes could occur in the same organisms. For example, *Lactococcus raffinolactis*, the Beijerinckiaceae bacterium RH AL1, and *Cyclotella* sp. accumulate vanadium, lanthanide, and chromium, respectively, in both the extracellular and intracellular compartments (Zhang et al. 2021; Wegner et al. 2021; Li et al. 2021). A few studies have reported that external factors induce a shift in the heavy metal distribution from one compartment to another. Al-Aoukaty et al. (1991) reported that lead accumulated intracellularly in phosphate-deficient medium and extracellularly in phosphate-rich medium. Cadmium was shown to accumulate mainly intracellularly in culture medium with lower metal concentrations and extracellularly in medium containing a higher cadmium concentration (Huang et al. 2014). Consistent with other studies, strains CD2-88 and CD2-102 showed a defense response to external factors by switching of the vanadium accumulation mechanism.

We hypothesized that vanadium-accumulating bacteria could help ascidians absorb vanadium in the intestine. The intestinal contents of *C. robusta* have pH 6–7 under physiological conditions, and in our study, CD2-88 and CD2-102 accumulated vanadium on the cell surface at pH 7 (Fig. 5). Under these conditions, most of the vanadium was reduced to V(IV) (see the subsection “Vanadium Speciation in Bacterial Cells” of the “Results” section). These results suggest that the bacteria took up V(V) from the intestinal contents and stored it in the extracellular compartment as V(IV), and could help ascidians absorb V(IV) via intestinal epithelial cells. These observations along with determination of the localization of metals provide insights into the interaction between ascidians and bacteria with regard to vanadium accumulation. A previous study on *Ascidia sydneiensis samea* reported that V(V) could be absorbed directly by strains V-RA-4 (*Vibrio tasmaniensis*) and S-RA-6 (*Shewanella kaireitica*) at the same pH (Romaidi and Ueki 2016). There may be different modes of interaction between microorganisms and ascidians in vanadium accumulation.

In summary, 12 bacterial strains were isolated from the intestinal contents of *C. robusta* and were classified into 8 genera. We identified two prospective bacterial strains with higher levels of vanadium accumulation, designated as CD2-88 and CD2-102. The accumulation and distribution of vanadium was pH-dependent, and nearly all of the accumulated V(V) was converted into V(IV). This research provided new insights into the symbiotic interactions between bacteria and *C. robusta*. The vanadium-accumulating and vanadium-reducing characteristics of both strains could be useful for application to bioremediation or biomineralization.

### Supplementary Information

Below is the link to the electronic supplementary material.

Supplementary file1 (XLSX 15 KB)

## Data Availability

Not applicable.
